# GeneXpert Based Confirmed Cases among Suspected Cases of Tuberculosis in a Tertiary Care Centre: A Descriptive Cross-sectional Study

**DOI:** 10.31729/jnma.7329

**Published:** 2022-03-31

**Authors:** Prabesh Pant, Kishor Gurung, Nitesh Shrestha, Subhechhya Basnet

**Affiliations:** 1 Department of Internal Medicine, Nepalgunj Medical College and Teaching Hospital, Nepalgunj, Banke, Nepal; 2 Department of Microbiology, Nepalgunj Medical College and Teaching Hospital, Nepalgunj, Banke, Nepal

**Keywords:** *multidrug-resistant*, *nucleic acid amplification test*, *pulmonary tuberculosis*

## Abstract

**Introduction:**

Tuberculosis is an infectious disease caused by Mycobacterium tuberculosis mostly affecting the lungs. Due to the low sensitivity of conventional microscopy and time-consuming culture method, Nucleic acid Amplification Assay Technique is preferred because of its rapidity and sensitivity. This test also helps in finding drug resistance to Rifampicin and also curtails the transmission of disease. The study is aimed to find the prevalence of GeneXpert confirmed cases among suspected cases of tuberculosis in a tertiary care centre.

**Methods:**

A descriptive cross-sectional study in 104 patients was conducted in a tertiary care centre from 30^th^ Dec 2021 to 3^rd^ Feb 2022. Ethical clearance was taken from the Institutional Review Committee (Reference number: 464/078/079). Sputum samples were collected from patients and were processed for GeneXpert under biological safety standards. GeneXpert Mycobacterium tuberculosis/rifampicin assay, sample processing, deoxyribonucleic acid extraction, and deoxyribonucleic acid amplification occurred in a fully automated cartridge-based real-time Polymerase chain reaction. A convenience sampling method was done. Collected data were coded as per variables and entered in Statistical Package for the Social Sciences version 25.0. Point estimate at 95% Confidence Interval was calculated along with frequency and percentage for binary data.

**Results:**

In all 104 patients, GeneXpert detected 10 (9.62%) (3.94-15.26 at 95% Confidence Interval) positive tuberculosis cases. Out of total positive cases, there were 6 (60%) males and 4 (40%) females and there was 1 (10%) rifampicin-resistant case.

**Conclusions:**

The prevalence of pulmonary tuberculosis among presumptive cases in our study was found to be similar to reported literature.

## INTRODUCTION

Tuberculosis is an infectious disease caused by Mycobacterium tuberculosis, which is a slender, straight or slightly curved bacillus with rounded ends.^1^ Pulmonary tuberculosis is primarily identified symptomatically using features like cough, fever, sweats, weight loss, hemoptysis, and extra-pulmonary lymph node swelling (lymphadenitis).^2^ Around 117,000 people with tuberculosis (TB) are living in Nepal.^3^ Among different diagnostic methods nucleic acid amplification (NAA) based (also known as GeneXpert) Mycobacterium tuberculosis/rifampicin (MTB/RIF) assay is sensitive and can simultaneously detect the presence of rifampicin resistance, which is a surrogate marker of multidrug-resistant (MDR) TB.^4^

According to a report of tuberculosis in the Southeast Asian region, in Nepal there has been a significant decrease in numbers of clinically diagnosed pulmonary tuberculosis cases than in previous years, this decrease is due to the introduction of Xpert assay as an initial confirmation test for TB.^5^

This study is aimed to find the prevalence of GeneXpert confirmed cases among suspected cases of tuberculosis in a tertiary care centre.

## METHODS

A descriptive cross-sectional study was conducted in Nepalgunj Medical College from 30^th^ Dec 2021 to 3^rd^ Feb 2022. A total of 104 (60 male and 44 female) patients were selected. Ethical clearance was taken from the Institutional Review Committee (Reference number: 464/078/079) from Nepalgunj Medical College. Histories of patients were taken and all the criteria were filled according to proforma. Before sample processing, informed consent was taken from patients and their relatives. The sample size was taken from the inclusion criteria of seven common symptoms as per the national tuberculosis guidelines met by the patients.^5^ The exclusion criteria include age less than 15 years, samples without clinical history, history of relapse and patients with a history of lung malignancies and fungal infection. In this study, convenience sampling was done.

The required sample size for this study was calculated using following formula:

n = (Z^2^ × p × q) / e^2^

  = (2.57^2^ × 0.066 × 0.934) / 0.02^2^

  = 97

Where,

n = minimum required sample size
Z = 1.96 at 95% Confidence Interval (CI)
p = prevalence of taken as 50% for maximum sample size
q = 1-p
e = margin of error, 10%

However, a total of 104 samples were taken in this study. Histories of patients were taken and all criteria were filled in a proforma. Data collection was done in a proforma data collection sheet and the results of the tests were recorded.

Sputum samples were collected in a tightly sealed leak-proof container. Patient name, hospital ID number, date, and time of collection were written on the sample container and carried to the microbiology laboratory. Sputum samples were collected from patients and samples were processed for GeneXpert under biological safety standards. GeneXpert MTB/RIF assay, sample processing, DNA extraction, and DNA amplification occurred in fully automated cartridge-based real-time PCR.

Collected data were coded as per variables and entered in Statistical Package for the Social Sciences version 25.0. Point estimate at 95% Confidence Interval was calculated along with frequency and percentage for binary data.

## RESULTS

A total of 104 respiratory specimens (sputum) are tested of which 10 (9.62%) samples are positive. The overall detection rate for both the male and female patients of all the age groups is 10 (9.62%) (Table 1). One (10%) patient from the age group of 45-59 years is found MTB/RIF resistant.

**Table 1:** Distribution of GeneXpert results in relation to age and sex (n= 10).

Age/Sex	Male		Female
	Positive n (%)		Positive n (%)
	**MTB/RIF resistance**		
15-29	1 (4.54)	-	1 (4.54)
30-44	1 (5.26)	-	-
45-59	1 (4.34)	1 (4.34)	1 (4.34)
60-74	2 (6.66)	-	1 (3.33)
75-90	-	-	1 (10)
Total	6 (60)		4 (40)

In this study, detection of MTB for male patients of age group 15-29 is 1 (4.54%), 30-44 is 1 (5.26%), 45-59 is 2 (8.69%), 60-74 is 2 (6.66%) whereas in female patients of age group 15-29 is 1 (4.54%), 45-59 is 1 (4.34%), 60-74 is 1 (3.33%) and 75-90 was 1 (10%).

In this study, GeneXpert positive cases are consistent with symptoms of cough for more than two weeks in 9 (90%), fever in 8 (80%), and weight loss with anorexia in 7 (70%) (Table 2).

**Table 2:** Observed symptoms (n= 104).

Symptoms	n (%)
Cough for more than two weeks	9 (90)
Fever with night sweats	8 (80)
Weight loss with anorexia	7 (70)
Hemoptysis	6 (60)
Extra-pulmonary lymph node swelling (lymphadenitis)	5 (50)
Fatigue	4 (40)
Shortness of breath along with chest pain	7 (70)

## DISCUSSION

In this study, we evaluated the GeneXpert confirmed cases in presumptive cases of pulmonary tuberculosis (PTB). The diagnosis time of GeneXpert is less than two hours so that it can detect PTB early and treatment can be initiated as soon as possible.^6^ Our study detected positive in 9.62% which is consistent with the study conducted in Ethiopia,^7^ but is less than the study conducted by Chinedum OK, et al. and Lombardi G, et al. where GeneXpert has a positive rate of (32.6-34.9%),^8^ and sensitivity of (83.7%) and a specificity of (99.1%) respectively.^9^ This difference may be due to the variation in the nature of study design, study setting, and sample size. In contrast to our study, there were other studies that showed a lower prevalence of TB among suspected cases 2.6%,^10^ and 4.6%." The low prevalence in comparison to our study might be due to variation in the study setting and study population.

In our study, the commonest symptom was cough for more than two weeks (90%) followed by fever (80%), and a history of weight loss with anorexia (70%) which is consistent with a study where cough and anorexia were importantly associated with a positive result in GeneXpert.^9^

Our study shows a high prevalence of rifampicin-resistant PTB (10%) compared with one study conducted in Ethiopia where they found rifampicin-resistant PTB in 5.3%.^7^ This difference may be due to the nature of the study population and sample size. However, in our study, rifampicin-resistant PTB is found in (10%) which is consistent with a similar study conducted in central Nepal (10.2%).^12^

Our study shows the GeneXpert detection more in males 6 (5.77%) than in females 4 (3.85%) which is consistent with the study conducted in central Nepal.^12^ In our study, age group 15-29 years in both male and female patients are affected more which is consistent with one study conducted by Rai DK, et al.^13^ where most of the cases affected were in between 15-30 years.

Studies have shown that smear-negative pulmonary tuberculosis is responsible for 13 to 17 percent of transmission.^13^ If early diagnosis is made using the GeneXpert test, it can decrease the rate of transmission of TB and its severity. As per the national tuberculosis guidelines, no patients should be trialled for TB treatment without bacteriological confirmation and all presumptive cases of pulmonary TB must have sputum examined.^4^ Also the national TB guidelines focus on the use of GeneXpert testing whenever there is possible access.

This study was conducted only on patients who visited the hospital with symptoms related to tuberculosis and the study would have been better if conducted on community-level along with radiographic evidence and sputum culture.

## CONCLUSIONS

The prevalence of PTB among presumptive cases in our study was found to be similar to published studies. GeneXpert is a sensitive and rapid test for diagnosing PTB in presumptive cases as well as helps in identifying Rifampicin resistance which is a surrogate marker of MDR-TB. The prevalence of GeneXpert positivity was seen more in male patients with the age group being most affected was between 15-29 years.
